# Thermostable Carbohydrate-Active Enzymes for High-Temperature Biomass Conversion: From Molecular Design to Process Integration

**DOI:** 10.3390/ijms27198788

**Published:** 2026-09-30

**Authors:** Yueli Hu, Xi Yang, Tianqi Wang, Mingshu Zheng, Zhenghao Jiang, Xinyue Dou, Xinkun Ren, Jiaxin Wu

**Affiliations:** 1 College of Engineering and Applied Sciences, Nanjing University, Nanjing 210023, China; 2 School of Food and Pharmaceutical Engineering, Nanjing Normal University, Nanjing 210023, China

**Keywords:** thermostable carbohydrate-active enzymes, high-temperature biomass conversion, design–build–test–learn (DBTL), starch liquefaction, lignocellulose saccharification, protein engineering

## Abstract

High-temperature processing of carbohydrate-rich biomass, including starch liquefaction and lignocellulose saccharification, can improve sugar yields, reduce contamination risks, and facilitate integration with thermochemical pretreatments. However, the operational stability of native carbohydrate-active enzymes (CAZymes) at 60–90 °C remains a major bottleneck for efficient and economically viable biorefineries, and existing reviews rarely integrate multiscale molecular mechanisms with process-relevant engineering strategies. This review systematically examines the multiscale basis of thermostability in CAZymes—covering core hydrophobic packing, electrostatic and metal-mediated networks, disulfide bonds, conformational dynamics, and modular architectures involving catalytic domains, linkers, and carbohydrate-binding modules—and directly links these features to enzyme longevity under high-solids, inhibitor-rich conditions. We then critically compare key engineering strategies, including structure-guided rational design, semi-rational directed evolution, consensus design, ancestral sequence reconstruction, and machine learning-assisted workflows embedded in design–build–test–learn (DBTL) cycles, highlighting their respective strengths, limitations, and complementarity. Representative case studies demonstrate that engineered thermophilic α-amylases and cellulases achieving ΔT_m_ improvements of approximately 10–20 °C can sustain >80–85% residual activity after prolonged exposure at process-relevant temperatures, translating into tangible benefits such as 15–30% reductions in enzyme dosing and measurable decreases in steam consumption, alongside higher sugar titers. By bridging molecular determinants, engineering paradigms, and quantitative process performance, this review provides a pragmatic roadmap for deploying thermostable CAZymes as robust biocatalysts in sustainable high-temperature biomass conversion.

## 1. Introduction

The global biorefinery sector—valued at approximately USD 176 billion in 2025 and projected to expand to USD 350–500 billion by 2035, representing a compound annual growth rate (CAGR) of ~8% [1,2]—is increasingly positioned as a foundational pillar of the circular bioeconomy, enabling the sustainable production of fuels, chemicals, and biomaterials from renewable carbohydrate feedstocks, including starch, cellulose, and hemicellulose [3]. Among the available process configurations, high-temperature bioprocessing (typically 60–90 °C) presents distinct operational advantages: reduced slurry viscosity, enhanced mass transfer kinetics, diminished risk of microbial contamination, and improved synergy with thermochemical pretreatment methods such as steam explosion and hydrothermal treatment [4,5]. These benefits collectively support higher solid loadings and volumetric productivities—key levers for improving techno-economic viability and reducing environmental impact per unit output. Nevertheless, widespread industrial adoption remains hindered by the thermo-functional instability of native carbohydrate-active enzymes (CAZymes), which frequently retain less than 20% of their initial activity after only a few hours under industrially relevant high-temperature, high-solids conditions [6,7,8].

CAZymes serve as catalytic gatekeepers that depolymerize recalcitrant polysaccharides into fermentable sugars and platform intermediates, thereby bridging renewable carbon feedstocks to downstream bioconversion pathways [9,10]. In starch processing, for instance, hydrothermal gelatinization followed by high-temperature liquefaction demands α-amylases capable of sustaining rapid endo-cleavage of swollen granules at 80–90 °C in highly viscous slurries—while preserving both a structurally ordered catalytic groove and a metal-ion-stabilized tertiary conformation [11,12]. In lignocellulose bioconversion, cellulase cocktails must act efficiently on insoluble, highly crystalline substrates amid lignin-derived inhibitors; this necessitates not only elevated melting temperatures (T_m_) but also multiscale functional stability—encompassing interdomain cooperation, linker-mediated flexibility, processive motility along cellulose chains, and sustained catalytic activity under industrially representative high-solids, inhibitor-rich conditions [7,13,14]. Field-scale demonstrations and techno-economic simulations consistently indicate that modest increases in optimal operating temperature (5–15 °C) and extensions of enzyme half-life yield substantial operational benefits: 15–30% reductions in enzyme loading, quantifiable decreases in steam consumption, and higher final sugar titers—all of which directly improve process economics and carbon efficiency [4,15,16,17].

Despite extensive work on individual thermostable enzymes and general concepts of protein thermostability, there is still a lack of an integrated, application-oriented framework that connects multiscale molecular determinants of CAZyme stability with modern engineering strategies and their consequences for high-temperature biomass conversion. Many biochemical reviews focus on structural stabilization mechanisms such as hydrophobic packing, electrostatic and metal-mediated networks, and disulfide bonds, but do not explicitly relate these features to enzyme performance under high-solids, inhibitor-rich environments [18,19]. Conversely, reviews on enzyme engineering and bioprocessing often emphasize rational design, directed evolution, or process intensification strategies without systematically analyzing how specific stability factors and modular architectures of CAZymes (catalytic domains, carbohydrate-binding modules, and linkers) contribute to operational robustness in starch liquefaction or lignocellulose saccharification [20,21,22,23]. Furthermore, the rapid emergence of machine learning-assisted protein design and synthetic biology-enabled thermophilic chassis has created new opportunities to tailor CAZymes for high-temperature biorefineries, but their potential and limitations have not yet been comprehensively assessed in the context of industrial biomass processing [24,25].

**Figure 1:**
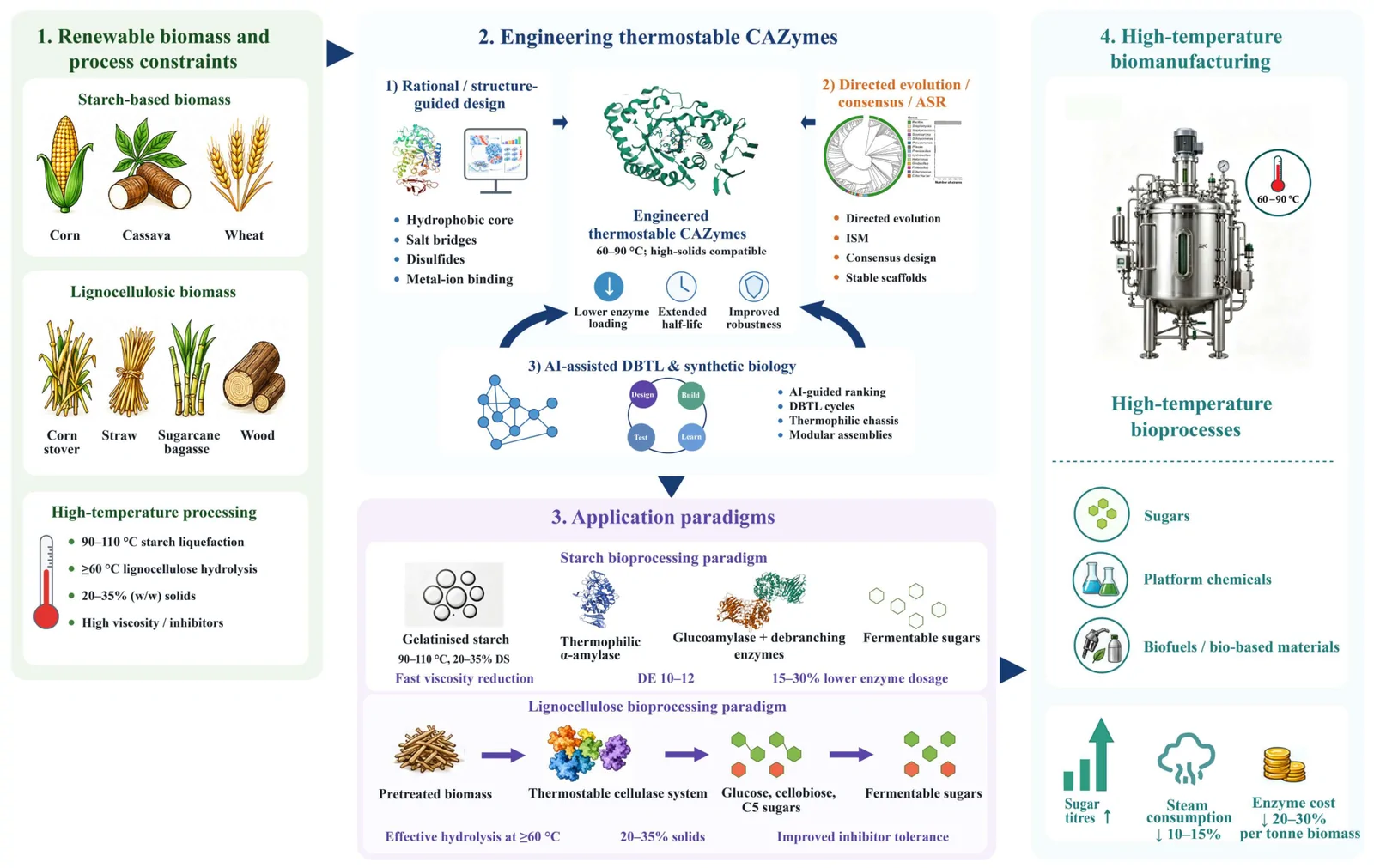
Thermostable CAZymes for high-temperature biomanufacturing: an overview. Renewable starch-based and lignocellulosic biomasses are processed under high-temperature, high-solids, and inhibitor-rich conditions, defining stringent operational windows for industrial CAZymes. Molecular features such as improved core packing, extended ion-pair networks and metal-ion binding provide baseline thermostability, which is further refined by rational design, directed evolution, consensus/ancestral approaches, AI-assisted design–build–test–learn cycles, and synthetic-biology-enabled chassis engineering. Engineered thermophilic amylases enable efficient starch liquefaction at 90–105 °C with reduced enzyme loading, while modular thermostable cellulase systems (endoglucanases, cellobiohydrolases, β-glucosidases and lytic polysaccharide monooxygenases (LPMOs)) operate at ≥60 °C on pretreated, high-solids lignocellulosic slurries. Integration of these enzymes and systems into thermophilic processes supports programmable high-temperature biomanufacturing of fermentable sugars, platform chemicals, and bio-based fuels/materials with improved sugar titers, lower steam consumption, and reduced enzyme cost per ton of biomass.

To bridge these gaps, this review provides a multiscale and translational overview of thermostable CAZymes for high-temperature biomass conversion. First, we summarize the thermodynamic and structural foundations of CAZyme thermostability, focusing on features that are directly relevant to enzyme longevity under process-like conditions, including core hydrophobic packing, electrostatic and metal-mediated interaction networks, conformational dynamics, and modular domain architectures involving catalytic domains, linkers, and carbohydrate-binding modules. Second, we critically evaluate major engineering strategies—structure-guided and semi-rational design, directed evolution, consensus design and ancestral sequence reconstruction, as well as machine learning-assisted workflows embedded in design–build–test–learn (DBTL) cycles—and discuss their respective strengths, limitations, and complementarity for generating robust CAZymes. Third, we highlight representative case studies of thermophilic α-amylases and cellulases deployed in high-temperature starch liquefaction and lignocellulose saccharification, illustrating how gains in T_m_, optimal temperature, and half-life translate into reduced enzyme usage, lower steam input, and improved sugar yields. Finally, we discuss how synthetic biology approaches and thermophilic microbial chassis can be used to integrate engineered CAZymes into consolidated bioprocesses and automated DBTL platforms. By explicitly linking molecular determinants, engineering paradigms, and process-level performance, this review aims to provide a practical roadmap for deploying thermostable CAZymes as robust biocatalysts in next-generation high-temperature biorefineries (Figure 1).

## 2. Molecular Basis of Thermostability in CAZymes for High-Temperature Biomass Conversion

### 2.1. Thermodynamic Framework and Process Relevant Stability

Protein thermostability essentially reflects whether the folded state can maintain an energetic advantage over the unfolded state as temperature increases [26]. For a reversibly folding protein, this process is commonly described by Gibbs free energy changes and the melting temperature, T_m_ [27]. As temperature rises, the free energy difference between the folded and unfolded states decreases, and the protein population gradually shifts from a native-state-dominated ensemble toward a larger unfolded-state fraction. Thus, T_m_ provides a basic thermodynamic measure of global folding stability.

In the context of biomass conversion, however, stability is not only a thermodynamic property but an operational performance metric. Carbohydrate-active enzymes (CAZymes) deployed in high-temperature starch liquefaction or lignocellulose saccharification must retain sufficient activity for hours at 60–90 °C, often in high-solids slurries and in the presence of inhibitors such as organic acids, phenolics, and high sugar concentrations. Industrially relevant stability is therefore better captured by residual activity profiles, half-life (t_½_) under process-like conditions, and robustness against aggregation and local unfolding at interfaces, rather than by T_m_ alone.

**Figure 2:**
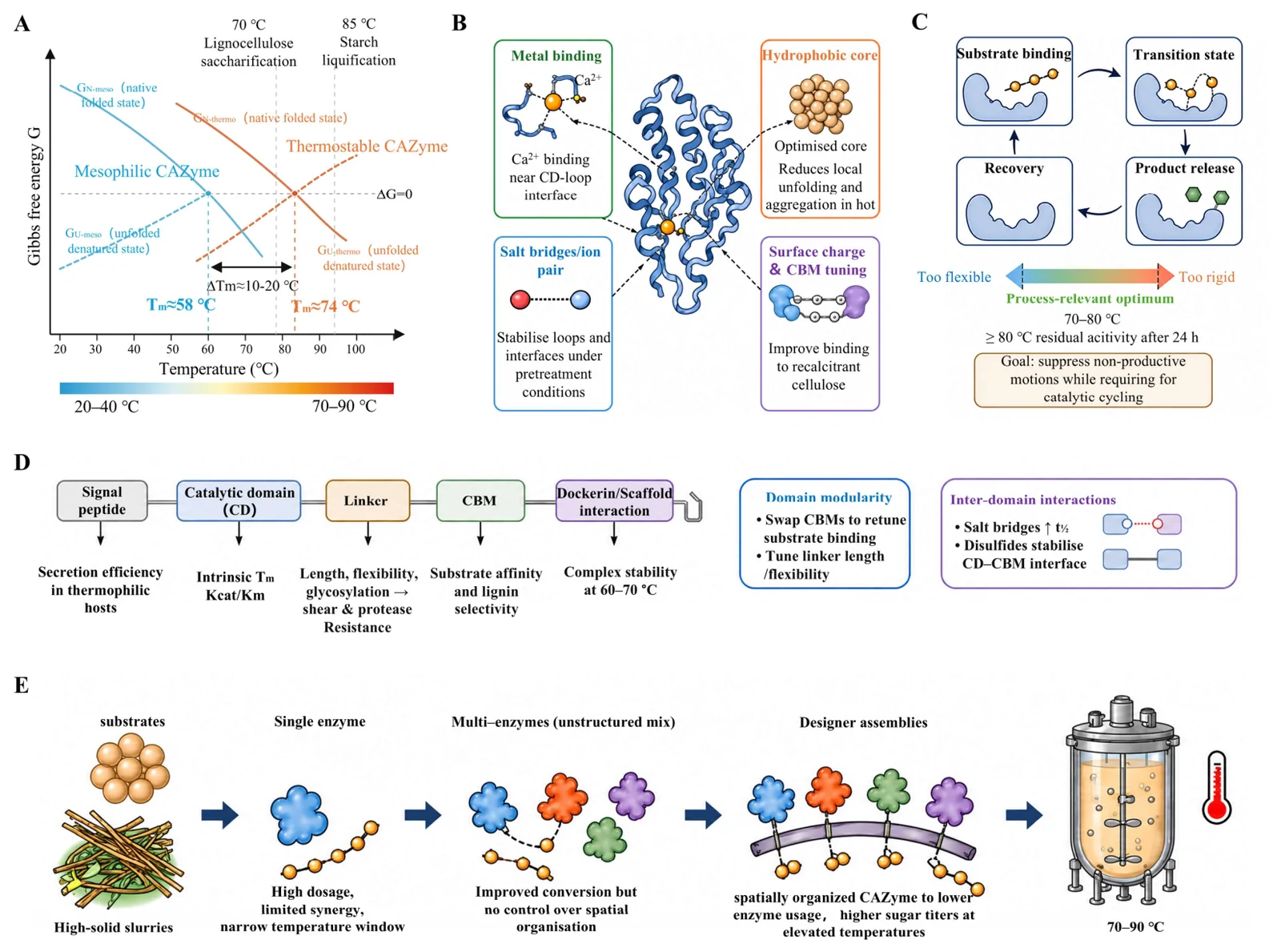
Multiscale molecular basis of CAZyme thermostability and its implications for high-temperature biomass conversion. (**A**) Thermodynamic stability landscapes comparing mesophilic and thermophilic CAZymes, aligned with process-relevant temperature windows (20–40 °C vs. 70–90 °C). (**B**) Key structural features that contribute to thermostability—optimised hydrophobic cores, electrostatic and metal-mediated networks, disulfide bonds, and tuned surface/carbohydrate-binding module (CBM) properties—illustrated in representative CAZyme scaffolds. (**C**) Conceptual view of the stability–activity trade-off during the catalytic cycle, highlighting the dynamic window that supports sustained activity under high-solids, inhibitor-rich conditions. (**D**) Modular CAZyme architecture showing how catalytic domains, linkers, CBMs, and dockerin/cohesin modules, together with inter-domain interactions, shape thermostability and process compatibility. (**E**) From single enzymes to multi-enzyme designer assemblies on lignocellulosic and starchy substrates, illustrating how spatial organisation and thermostability enable reduced enzyme loading, lower steam consumption, and higher sugar yields in high-temperature bioprocesses.

Thermophilic proteins typically reshape the folding energy landscape at elevated temperatures through more favourable interaction networks in the folded state, a more restricted conformational space in the unfolded state, or altered heat-capacity effects [27,28]. Stabilizing factors such as salt bridges, hydrophobic packing, hydrogen-bond networks, disulfide bonds, and metal binding can therefore be viewed as local adjustments to this energy landscape, rather than isolated structural features. For CAZymes used in biorefineries, the key question is whether these energetic gains are effectively translated into sustained catalysis under high-temperature, high-solids, inhibitor-rich conditions. The following sections discuss how specific structural and dynamic features support such operational stability (Figure 2A).

### 2.2. Structural Determinants of Thermostability with Clear Process Impact

The structural basis of thermostable CAZymes is mainly reflected in stabilizing elements such as hydrophobic cores, salt bridges and surface electrostatic networks, disulfide bonds, and metal binding. By comparing 18 pairs of thermophilic proteins and their mesophilic homologs, Kumar et al. found that increases in salt bridges and side-chain–side-chain hydrogen bonds represented relatively consistent trends, whereas overall hydrophobicity, close packing, main-chain hydrogen bonding, and oligomeric state did not show uniform changes [29]. This indicates that thermostability depends more strongly on local interaction networks within specific folding contexts [27,30]. In CAZymes, these stabilizing elements are often distributed in conserved folding cores, substrate-binding grooves, flexible loops, domain interfaces, and metal-binding regions.

The hydrophobic core mainly maintains the overall folding framework. Kumar and Nussinov suggested that some thermophilic proteins enhance stability through increased branched hydrophobic residues, improved core packing, and reduced internal cavities [31]. These features help reduce the risk of water penetration and local unfolding in the protein core at elevated temperatures. In CAZymes, this type of stabilization is often reflected in conserved catalytic scaffolds, such as triosephosphate isomerase (TIM) barrels and β-jelly-roll folds. However, stronger hydrophobicity is not necessarily better. The comparative analysis by Kumar et al. also showed that overall hydrophobicity is not a universally increased factor [29]. Therefore, hydrophobic engineering should be understood more as local packing optimization than as a general increase in hydrophobic character.

Elcock showed that salt bridges may contribute to stability at high temperature by restricting conformational fluctuations [32]. Lee et al. further validated the engineering potential of salt-bridge design in β-glucosidase [33]. In their study, eight candidate salt bridges were designed and 24 mutants were constructed. Single-point mutations increased T_m_ by 0.7–8.8 °C, while combined mutations increased T_m_ by up to 15.7 °C. This demonstrates that salt bridges are not only common structural features in thermophilic proteins, but also engineerable stabilizing elements. In CAZymes, salt bridges and electrostatic networks are often located on protein surfaces, near substrate-binding grooves, at domain interfaces, or around thermally sensitive loops.

Disulfide bonds provide covalent constraint-based stabilization. Vieille and Zeikus noted in their summary of hyperthermophilic enzymes that high thermostability is usually not determined by uniform global reinforcement, but rather by a limited number of specific structural changes [34]. Disulfide bonds represent this type of stabilizing element, as they can strongly restrict local conformational freedom and are often used to stabilize flexible loops, terminal regions, or domain-connecting regions. In CAZymes, their value lies not simply in adding covalent linkages, but in reducing local structural fluctuations at high temperature. However, if introduced at inappropriate positions, disulfide bonds may also disrupt native folding or catalytic conformation.

Metal binding stabilizes specific structural regions through coordination interactions. Metal ions such as Ca^2+^ and Na^+^ can bridge distant residues, neutralize local charges, and induce disordered regions to become ordered [19]. In many α-amylases, Ca^2+^ or Ca–Na–Ca sites are located near domain interfaces or substrate-binding grooves, where they stabilize local folding and maintain specific conformational states. Therefore, metal ions often function as part of the structural stabilization network in CAZymes.

Overall, the structural basis of thermostable CAZymes results from local interaction networks. The comparative study by Kumar et al. indicates that among the various structural parameters, only salt bridges and side-chain–side-chain hydrogen bonds show relatively consistent increases, whereas other factors are strongly context-dependent. Hydrophobic cores, electrostatic networks, disulfide bonds, and metal binding shape high-temperature structural stability through core packing, surface interactions, covalent constraints, and coordination stabilization, respectively [27]. These features also provide a structural foundation for subsequent thermostability engineering (Figure 2B).

### 2.3. Conformational Dynamics and the Stability–Activity Trade-Off

Thermostability is not determined solely by static structure, but is also strongly influenced by conformational dynamics. During catalysis, enzymes undergo a series of conformational changes, including substrate binding, active-site rearrangement, transition-state stabilization, and product release [18], as illustrated in Figure 2C. Therefore, a completely rigid protein structure does not necessarily correspond to higher functional stability. Eijsink et al. showed in their review of enzyme stability engineering that thermal inactivation is often not caused by simple reversible global unfolding, but rather by local unfolding, aggregation, or irreversible conformational damage [35]. This implies that the goal of thermostability engineering is not to suppress all protein motions or simply increase structural rigidity, but to distinguish which motions lead to inactivation and which are required for catalysis, and then optimize them in a balanced manner.

The relationship between stability and activity is neither strictly negative nor strictly positive. The concept of protein dynamism proposed by Tokuriki and Tawfik emphasizes that proteins exist as ensembles of multiple conformational states, and that moderate structural fluctuations provide the basis for substrate recognition, functional plasticity, and evolutionary adaptability [36,37]. Bloom et al. further demonstrated in P450 BM3 that increased stability can enhance mutational robustness, allowing enzymes to tolerate otherwise destabilizing functional mutations and acquire new substrate activities [38]. Thus, stability does not inherently limit function; when stabilization occurs in non-productive flexible regions or structurally vulnerable elements, it may instead provide a larger mutational and functional buffer. The key issue is where stability is introduced. If stabilizing mutations occur in regions required for catalytic motions, substrate access, or product release, activity may decrease. In contrast, if they restrict local unfolding, slow conformational exchange, or aggregation-prone regions away from the active site, they can simultaneously improve thermostability and functional retention. Hou et al. showed in a ubiquitin study that millisecond-timescale conformational exchange is correlated with thermal stability, and that alanine substitutions in flexible regions can significantly enhance stability [19]. This suggests that thermal inactivation is often associated with specific dynamic regions rather than global structural loosening.

For CAZymes, this dynamic trade-off is particularly important. Amylases must maintain the geometry of the substrate-binding groove, metal coordination network, and catalytic residues while still allowing substrate entry and product release. Cellulases, in contrast, require an active-site tunnel, processive motion along cellulose chains, CBM positioning, and linker flexibility. Excessive rigidification may restrict substrate accessibility, chain sliding, or processive hydrolysis, whereas excessive flexibility can accelerate local destabilization at high temperature. Therefore, the key objective of CAZyme thermostability engineering is to suppress non-productive motions that lead to inactivation while preserving functional dynamics associated with substrate processing and catalytic cycling [18,19,35].

### 2.4. Thermostability in Modular CAZyme Architectures

Many CAZymes are modular proteins composed of a catalytic domain, a carbohydrate-binding module (CBM), and a linker [39,40,41]. For these enzymes, thermostability is not only determined by the folding stability of the catalytic domain, but also by the maintenance of spatial organization and dynamic coupling among different modules. The CBM is responsible for substrate targeting, the linker defines the distance and mobility between the catalytic domain and the CBM, and the catalytic domain performs glycosidic bond hydrolysis [40,42]. The relative positioning and coordinated interactions among these three components collectively determine whether modular enzymes can maintain effective function at elevated temperatures.

The primary role of CBMs is to enhance effective contact between enzymes and polysaccharide substrates. Structural studies have shown that CBMs mediate polysaccharide recognition through binding surface geometry, aromatic residue platforms, and hydrogen-bond networks [40,43]. Boraston et al. described CBMs as modules for fine-tuning polysaccharide recognition, while structural reviews by Hashimoto et al. further demonstrated that different CBM families achieve selective carbohydrate recognition through compact folds and specialized binding interfaces [40,43]. Thus, CBM stability not only affects whether the module itself remains properly folded, but also influences the positioning and orientation of the catalytic domain at the reaction interface. The linker provides spatial accessibility and conformational flexibility between modules. Its length, amino acid composition, and glycosylation state influence the distance, relative motion, and proteolytic resistance between the catalytic domain (CD) and CBM [13,42]. A linker that is too short may restrict access of the catalytic domain to substrate chains, whereas an excessively long or flexible linker may weaken interdomain coupling. Therefore, the linker is a key structural element that regulates both conformational dynamics and functional stability in modular CAZymes.

Modular architecture also provides independent levels for thermostability engineering. He et al. fused a thermostable CBM A2 from *Thermotoga maritima* XynA to the N-terminus of *Trichoderma reesei* xylanase 2 [37]. As a result, the optimal temperature of the chimeric enzyme increased from 55 °C to 65 °C, and more than 85% of its activity was retained after 30 min at 60 °C [37]. This case demonstrates that module swapping or heterologous fusion can not only alter substrate-binding properties, but also improve functional stability at high temperature by enhancing module robustness and interdomain organization.

In essence, the thermostability of modular CAZymes reflects coordinated module stability. The CBM, linker, and catalytic domain are responsible for substrate targeting, structural connection, and catalysis, respectively, but their stabilities are not independent [13,40,41]. Only when each module is stable, their spatial arrangement is properly maintained, and their conformational dynamics remain balanced, can modular CAZymes sustain efficient catalysis under high-temperature conditions. The modular organization of CAZymes and the contributions of individual domains and interdomain interactions are summarized in Figure 2D.

### 2.5. From Protein Stability to System Compatibility

The molecular, dynamic, and modular foundations of CAZymes are critical for their thermostability. However, in real reaction systems, a stable protein does not necessarily translate into an efficient catalytic module. Parameters such as T_m_, T_50_ (the temperature at which 50% of the initial activity remains under a specified assay or heat-treatment protocol), or short-term residual activity describe the response of proteins to temperature elevation or heat treatment, but they do not fully capture the sustained performance of enzymes under target substrates, long reaction times, and complex chemical environments [44,45]. Therefore, the applicability of CAZymes at high temperatures should be extended from protein stability to system compatibility.

A higher T_m_ may indicate enhanced resistance to global unfolding, but it does not account for irreversible inactivation pathways or the operational stresses encountered during prolonged biomass conversion. At high substrate loadings, increased viscosity, restricted mass transfer, limited water availability, and nonproductive adsorption can reduce effective catalytic turnover even when the enzyme remains folded. Pretreatment-derived phenolics and organic acids, accumulated sugars, salts, and other inhibitors may interfere with substrate binding or accelerate enzyme inactivation, whereas changes in pH can alter the ionization states of catalytic residues, surface charge, salt bridges, and metal-binding networks. Consequently, process performance should be evaluated using a combination of T_m_, inactivation half-life, residual activity, and substrate conversion under defined temperature, pH, solids loading, inhibitor composition, and reaction time.

System compatibility is mainly reflected at three levels. First, enzymes must maintain effective binding, substrate accessibility, and continuous catalysis on target substrates at high-solids loadings [44,45,46]. Second, enzymes must tolerate process-related chemical conditions such as pretreatment-derived inhibitors, product accumulation, salts, pH variation, or solvents [47,48,49]. Third, the stability of individual enzymes must be compatible with other CAZyme components to achieve overall efficiency in multienzyme systems [46,50]. Østby et al. emphasized in their analysis of lignocellulosic saccharification that efficient conversion depends on the compatibility among feedstock type, pretreatment method, enzyme cocktail composition, and reaction process [51]. Similarly, Wolski et al. showed in their review of ionic liquid systems that pretreatment chemicals can simultaneously affect both the activity and stability of biomass-degrading enzymes. These studies indicate that thermostability has practical relevance only when it is maintained within system-level constraints [47].

Taken together, CAZyme thermostability should be viewed as a multilevel system property. Folding stability, functional dynamics, modular coordination, substrate compatibility, chemical tolerance, and multienzyme synergy collectively determine catalytic performance under high-temperature conditions. This perspective also provides a foundation for subsequent engineering strategies: the goal of thermostable CAZyme engineering is to translate molecular-level stability into sustained, coordinated, and operational enzymatic function under target process conditions [44,50]. The progression from individual enzymes to multienzyme assemblies under process-relevant conditions is illustrated in Figure 2E.

## 3. Engineering Strategies for Thermophilic CAZymes in High-Temperature Bioprocessing

For biorefineries operating at 60–90 °C and high-solids loadings, the central engineering objective is not only to increase the melting temperature (T_m_) of carbohydrate-active enzymes (CAZymes), but to achieve long operational half-lives and sustained catalytic performance under process-relevant conditions, thereby reducing enzyme loading, steam consumption, and the overall production costs. This section discusses major strategies for engineering thermophilic CAZymes—structure-guided rational design, directed evolution, consensus design and ancestral sequence reconstruction, AI-guided workflows, and synthetic-biology-enabled integration—with emphasis on their implications for high-temperature starch processing and lignocellulose conversion.

### 3.1. Rational Design: Structure-Guided Thermostability Engineering

**Figure 3:**
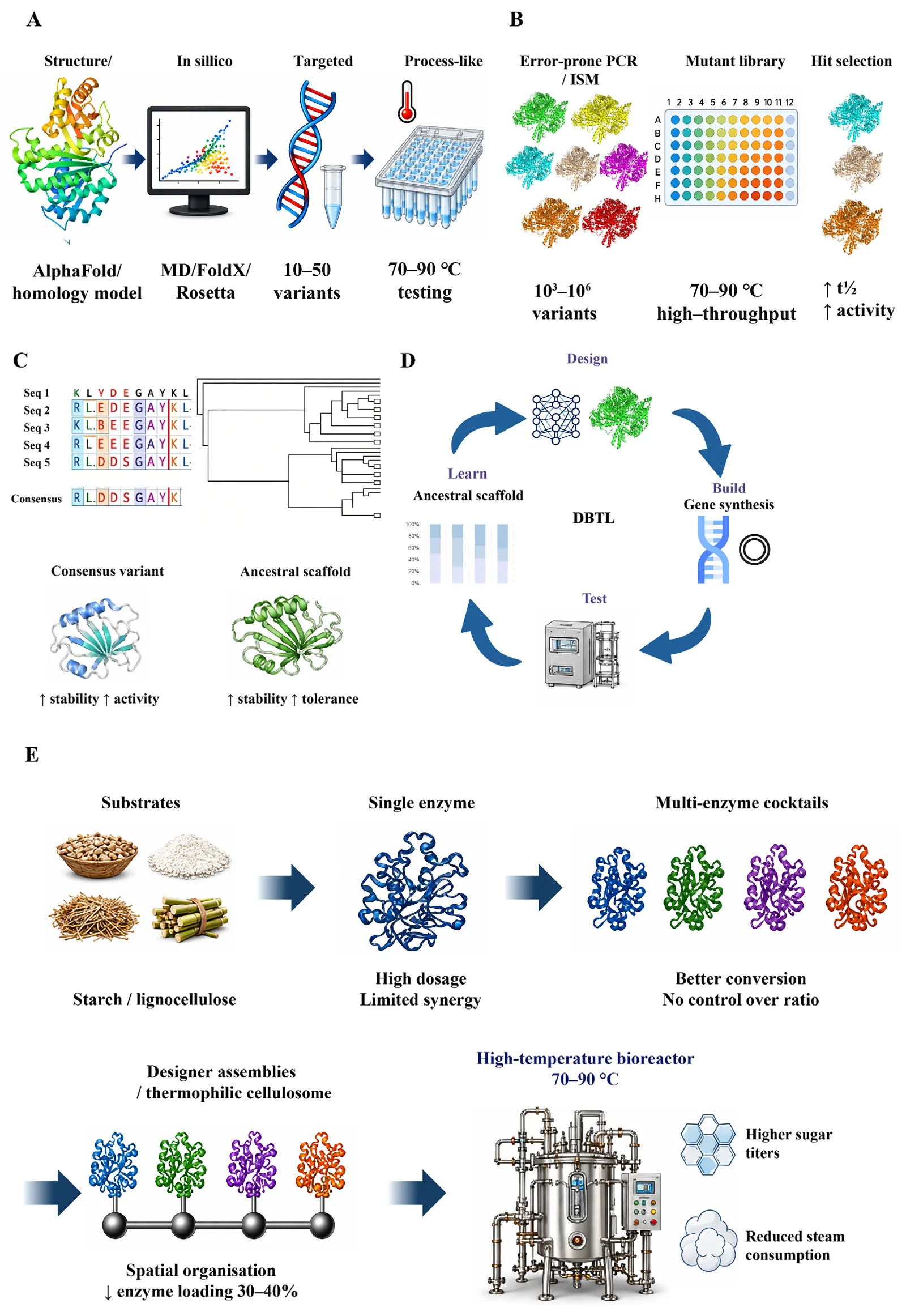
Engineering strategies for thermostable CAZymes in high-temperature bioprocessing. (**A**) Structure-guided rational design workflow for identifying thermally sensitive regions, predicting stabilizing mutations, and validating a small number of variants under process-like conditions. (**B**) Directed evolution and high-throughput screening strategies that enrich CAZyme variants retaining activity in high-temperature, high-solids, inhibitor-rich media. (**C**) Consensus design and ancestral sequence reconstruction provide evolution-guided, highly stable scaffolds for subsequent optimization. (**D**) AI-assisted design–build–test–learn (DBTL) cycles integrate structural modelling, machine learning, and experimental feedback to efficiently explore sequence space under process constraints. (**E**) Synthetic-biology-enabled integration from single enzymes to multi-enzyme cocktails and thermophilic designer assemblies, enabling reduced enzyme loading and improved sugar yields in high-temperature starch and lignocellulose bioprocesses.

Rational design is a structure- and mechanism-based directed engineering approach. Unlike random mutagenesis, it first identifies where an enzyme is likely to become unstable under high-temperature conditions, and then designs a small number of candidate mutations targeting these regions for experimental validation. A typical workflow includes four steps: obtaining a protein structure or a reliable model, identifying potential thermally sensitive regions, selecting appropriate stabilization strategies, and experimentally evaluating the thermostability and catalytic performance of the resulting variants. The rational-design workflow is illustrated in Figure 3A.

Rational design can rely on multiple types of information. Structural information is used to identify internal cavities, loose packing, flexible loops, exposed hydrophobic patches, or unstable interfaces [52]. Sequence conservation and consensus analysis help determine whether a given position tends to be occupied by more favorable residues in stable homologs. Molecular dynamics simulations can identify locally flexible regions with large fluctuations at elevated temperatures. Computational tools such as FoldX, Rosetta, FireProt, and Protein Repair One-Stop Shop (PROSS) can further predict the effects of mutations on folding free energy, structural constraints, and combinatorial interactions [53,54]. The common goal of these approaches is to narrow the vast mutational space into a small number of interpretable and experimentally testable candidates. In practical engineering, rational design typically employs several stabilization strategies. Hydrophobic core repair is used to fill internal cavities or improve packing. Salt-bridge and hydrogen-bond engineering enhance surface or interfacial interactions. Disulfide bond introduction restricts excessive fluctuations in loops or terminal regions at high temperature. Truncation of flexible segments removes redundant regions that may trigger local instability. The choice of strategy depends on the structural weakness of the target region, rather than simply adding stabilizing interactions to the protein.

Several CAZyme engineering cases demonstrate the effectiveness of this approach. Lu et al. engineered a GH11 xylanase from *Paecilomyces thermophila* by integrating folding free energy calculations, evolutionary analysis, and molecular dynamics screening of mutation combinations [55]. The resulting variant XynM9 showed an increased optimal temperature of 85 °C, a T_m_ of 93 °C, and an extended half-life of 5.8 h while also exhibiting improved catalytic efficiency. Structural analysis revealed increased hydrogen bonds and salt bridges, along with a narrowed catalytic cleft that enhanced substrate binding. This indicates that when rational design targets are closely related to both structural stability and substrate recognition, improvements in thermostability and catalytic performance can be achieved simultaneously. Flexible region engineering further illustrates the logic of rational design. Yang et al. truncated the N- and C-termini of a GH11 xylanase, obtaining the 28C variant, which exhibited increased catalytic activity, a higher optimal temperature, and improved residual activity after treatment at 50–65 °C [56]. This suggests that redundant flexible segments in terminal or loop regions may serve as initiation points for thermal destabilization, and that appropriate truncation or rigidification can enhance thermostability. Similarly, disulfide bond engineering and salt-bridge design are most effective when they stabilize thermally sensitive regions or reinforce key structural interfaces, and are more likely to result in improvements in T_m_, half-life, or residual activity.

Overall, the strength of rational design lies in its low mutation burden, clear mechanistic logic, and interpretability, making it an effective starting point for CAZyme thermostability engineering. It enables the rapid generation of candidate designs through structural analysis and computational prediction, but cannot fully replace experimental screening, as the effects of mutations on activity, expression, substrate compatibility, and long-term stability are often difficult to predict accurately. Therefore, rational design is best used in combination with small mutational libraries, directed evolution, or high-throughput screening to balance interpretability and experimental efficiency [35].

### 3.2. Directed Evolution and High-Throughput Screening

Directed evolution is a protein engineering approach that generates a mutant library and applies selection pressure to enrich variants with desired phenotypes over iterative rounds. In contrast to rational design, which relies on structural hypotheses, directed evolution does not require a complete understanding of thermal inactivation mechanisms in advance. Instead, it allows the experimental system to directly select variants that retain function under high-temperature conditions. The concepts of deoxyribonucleic acid (DNA) shuffling proposed by Stemmer, enzyme directed evolution developed by Arnold, and family shuffling introduced by Crameri et al. established this framework, in which beneficial mutations can accumulate through iterative rounds of mutagenesis and selection, while functional segments from homologous sequences can be recombined to generate new and more stable variants [20,57,58,59].

For thermostable CAZymes, the value of directed evolution lies in its ability to explore unpredictable mutation combinations [60]. Many stabilizing mutations exhibit non-additive effects: individual mutations may have limited impact, but their combinations can significantly enhance stability. Conversely, some seemingly beneficial mutations may reduce activity or expression. The iterative saturation mutagenesis (ISM) strategy proposed by Reetz and Carballeira provides a more focused approach by first selecting a limited number of key residues based on structural or functional information, followed by saturation mutagenesis and iterative recombination to identify variants with balanced stability and activity [61]. This semi-rational strategy reduces library size and is particularly suitable for optimizing regions such as catalytic grooves, substrate-entry loops, metal-binding sites, and domain interfaces in CAZymes.

The screening system determines the outcome of directed evolution. As emphasized by Packer and Liu in their overview of directed evolution, the mutant library only provides diversity, while the screening or selection method ultimately defines success by capturing the desired phenotype [62]. For thermostability engineering, screening typically focuses on residual activity after heat pretreatment, catalytic rates at elevated temperatures, or colorimetric/fluorometric assays following thermal challenge. High-throughput methods such as microplate screening, fluorescence-activated cell sorting (FACS), microdroplet systems, and deep mutational scanning are widely used to increase throughput and improve phenotypic resolution [63,64,65]. If screening focuses only on T_m_ or room-temperature activity, it may fail to identify variants with sustained catalytic performance under process-relevant conditions. Therefore, screening pressure for CAZyme engineering should simultaneously reflect stability and functional retention.

Overall, directed evolution and high-throughput screening provide an experimental approach to explore mutations and identify thermostable CAZyme variants [20,60]. Its core strength does not lie in fully explaining each mutation, but in identifying sequences that retain effective catalytic function under high-temperature conditions through appropriate library design and selection pressure. Compared with rational design, directed evolution is more suitable for cases where mechanisms are unclear, mutation effects are difficult to predict, or stability–activity trade-offs are complex [62]. The workflow of library generation and iterative screening is shown in Figure 3B.

### 3.3. Consensus Design and Ancestral Sequence Reconstruction

Rational design usually starts from local weaknesses in existing enzymes, whereas directed evolution identifies beneficial mutation combinations through screening. Both strategies, however, share an implicit assumption: the existing enzyme scaffold is still suitable as an engineering starting point. For some CAZymes with limited stability margins, this assumption may not hold. In such cases, evolutionary information provides an alternative route (Figure 3C). Homologous sequence statistics or phylogenetic history can be used to recover scaffolds with higher intrinsic stability and greater engineering tolerance [66,67].

Consensus design is an early representative of this strategy. Steipe et al. showed that sequence statistics can predict some stabilizing mutations, suggesting that residues repeatedly observed in homologous sequences often encode folding stability or structural constraints [68]. Lehmann et al. applied the consensus concept to protein thermostability engineering and substantially improved the thermostability of fungal phytase by constructing a consensus sequence [69]. This demonstrated that protein stability can be reshaped at the sequence level. A key advantage of consensus design is that it does not fully depend on high-resolution structural information, making it suitable for enzyme families with abundant homologous sequences but limited structural data [66]. However, consensus mutations are not necessarily stabilizing. Sullivan et al. further showed that site conservation and inter-residue correlations jointly influence mutation outcomes [70]. Ignoring coevolutionary relationships may introduce residues that do not match the local structural context. Thus, consensus design is better viewed as a source of evolutionary constraints rather than a simple replacement of target residues with the most frequent amino acids.

Ancestral sequence reconstruction (ASR) further places sequence information into an evolutionary-history framework. Unlike consensus design, which extracts statistical averages from modern sequences, ASR infers sequences at specific ancestral nodes using phylogenetic trees and substitution models, followed by gene synthesis and experimental characterization of the resurrected proteins [66,67]. Gaucher et al. used resurrected proteins to infer ancient temperature trends in Precambrian life, showing that ancestral proteins can exhibit high thermostability [71]. Hobbs et al. reconstructed ancestral *Bacillus* LeuB enzymes and showed that thermal adaptation did not follow a simple linear trajectory, but fluctuated with lineage history [72]. Akanuma et al. reconstructed ancestral nucleoside diphosphate kinases (NDKs) with extremely high thermostability, providing experimental support for the thermophilic nature of early life [73]. Risso et al. resurrected Precambrian β-lactamases with both high stability and broad substrate adaptability, further indicating that ancestral proteins may provide not only enhanced stability, but also greater functional plasticity [74].

For CAZymes used in high-temperature biomanufacturing, enzymes often need to withstand high temperatures, complex substrates, long reaction times, and destabilizing mutations introduced during later activity optimization. If the starting template has insufficient stability, mutations aimed at altering substrate specificity, catalytic efficiency, or modular architecture can easily compromise folding. The value of consensus design and ASR therefore lies in providing starting platforms with larger stability margins, allowing subsequent rational design, directed evolution, or modular recombination to proceed with greater tolerance [66,67].

Nevertheless, evolution-guided scaffolds are better regarded as engineering starting points rather than final optimized enzymes, and should be combined with structural design and experimental screening. For thermostable CAZyme development, a stable and evolvable scaffold is often more important than a single highly stabilizing mutation because it determines how far subsequent optimization of activity, substrate adaptation, and process compatibility can proceed [67].

### 3.4. AI-Guided Thermostability Engineering

Traditional protein engineering often proceeds through a linear workflow of proposing a small number of mutations, experimentally validating them, and then performing further optimization. Machine learning and automated platforms are now turning design, build, test, and learn into an increasingly closed-loop process (Figure 3D) [25,75,76]. Although the automated DBTL workflow proposed by Carbonell et al. was mainly developed for metabolic pathway optimization, its core logic is also applicable to enzyme engineering: experimental data are no longer used only for validation, but are fed back to train models and guide the next round of design [77]. For thermostable CAZymes, this means that engineering can move beyond a single screen for high-temperature activity toward continuous learning of the relationships among sequence, structure, stability, substrate adaptation, and process performance [54].

Machine learning first improves the efficiency of mutational-space exploration [78,79,80]. Yang, Wu, and Arnold described machine-learning-guided directed evolution as a new paradigm for protein engineering, in which measured variants are used to train sequence–function models that can then predict more promising candidate sequences [81]. Biswas et al. further demonstrated that even with limited experimental data, low-N deep learning can be used to build virtual fitness landscapes and screen large numbers of sequences computationally [82]. These methods are particularly valuable for CAZymes because many realistic screening conditions are difficult to implement in high-throughput formats, such as the hydrolysis of insoluble polysaccharide substrates, long-term reactions at elevated temperatures, high-solids systems, and the presence of inhibitors. The value of AI lies in reducing unproductive mutational searches and focusing limited experimental resources on candidate sequences with higher information content.

Structure prediction models have also greatly lowered the barrier to the rational analysis of CAZymes. AlphaFold and RoseTTAFold have made it possible to model, compare, and design many glycoside hydrolases, CBMs, LPMOs, and modular CAZymes that lack crystal structures [22,83,84]. Protein language models developed by Rives et al. and Lin et al. further indicate that large-scale protein sequence data contain structural and functional constraints that can be learned by models [24,85,86]. For thermostable CAZyme engineering, these models can help identify potential thermally sensitive regions, construct mutation libraries, analyze modular interfaces, or predict structural frameworks of distant homologs. However, predictable structure does not necessarily mean predictable function. The actual performance of CAZymes also depends on substrate surfaces, processive motion, intermodule dynamics, multienzyme synergy, and process environments, which are often beyond the descriptive capacity of a single static structural model.

Generative models further extend protein engineering from modifying existing enzymes to designing new sequences and scaffolds. The protein language model ProtGPT2 can generate new sequences with natural protein-like features; RFdiffusion has demonstrated the ability to generate protein scaffolds under structural or functional constraints; and ESM3 further integrates sequence, structure, and function into a unified multimodal generative framework [24,87,88,89]. These advances open new possibilities for thermostable CAZyme engineering. Future design may no longer be limited to point mutations in existing enzymes, but may increasingly focus on the generation of stable scaffolds, loop reconstruction, modular-interface optimization, and even new combinations of CBMs, linkers, and catalytic domains. However, current generative AI is still better at ensuring that sequences appear protein-like and structures are foldable, while catalytic efficiency on real polysaccharide substrates, processive hydrolysis, and long-term stability still require experimental validation.

Recent advances in active learning and autonomous protein engineering further indicate that AI is moving from passive prediction toward experimental decision-making [75,79,90,91,92]. Active learning can prioritize the most informative variants for the next round of testing, thereby improving sequence-space exploration when epistatic effects are present [79,80]. This is particularly relevant to thermostable CAZyme engineering, where thermostability, catalytic efficiency, expression, substrate compatibility, and inhibitor tolerance often need to be optimized simultaneously [54,93]. In this context, the value of AI is not simply to predict more mutations, but to propose a small number of experimentally testable and mechanistically interpretable candidates [25,91]. For high-temperature biomanufacturing, future AI models should go beyond structure-aware prediction and incorporate data from real-substrate hydrolysis, long-term high-temperature reactions, multienzyme systems, and process conditions [54,93]. Only by connecting sequence–structure–function relationships with process-level constraints can AI-guided design convert thermostable CAZymes from computational candidates into practical biomanufacturing modules [25,80].

### 3.5. Synthetic Biology-Enabled Thermophilic Engineering

As rational design, directed evolution, ancestral reconstruction, and AI-assisted design continue to expand the accessible sequence space, the bottleneck in thermostable CAZyme engineering is gradually shifting from how to propose mutations to how to rapidly build, test, and iterate them. This is where synthetic biology becomes particularly important. By integrating otherwise separate design strategies into the Design–Build–Test–Learn (DBTL) cycle, synthetic biology transforms thermostability engineering from case-by-case optimization into an iterative, scalable, and data-driven engineering process.

In the Design stage, stabilization hypotheses can be derived from structural and mechanistic analysis, evolutionary conservation, ancestral sequence reconstruction, machine-learning models, or comparative genomics. In the Build stage, gene synthesis, modular cloning, multisite mutagenesis, and genome editing can be used to rapidly construct variant libraries, domain-recombined enzymes, or engineered strains. In the Test stage, screening readouts should not be limited to T_m_ or short-term residual activity, but should also include more application-relevant phenotypes, such as activity retention after heat treatment, conversion of target substrates, secretion level, and tolerance to process-related chemical environments. In the Learn stage, experimental data are fed back into the next round of design to refine models and narrow the search space. The development of automated DBTL platforms and biofoundries has demonstrated that standardization, automation, and data-driven workflows can greatly improve the efficiency of biological system construction and testing [25,75,76].

Synthetic biology also provides modular construction capabilities for CAZyme engineering. Many CAZymes are naturally composed of catalytic domains, CBMs, linkers, signal peptides, or auxiliary domains, making them suitable for combinatorial design through domain recombination, element replacement, and expression-system optimization. Unlike simple point mutation, modular construction can simultaneously tune enzyme stability, substrate targeting, secretion, and system compatibility. For thermostable CAZymes, this means that the engineering target is no longer limited to individual amino acid residues, but can also include domain combinations, expression elements, enzyme ratios, and host environments [94,95,96].

Thermophilic or thermotolerant chassis further expand the application space of thermostable CAZymes. Conventional strategies often express thermostable enzymes in mesophilic hosts and then apply the purified enzymes in high-temperature in vitro reactions. In contrast, thermophilic microorganisms such as *Clostridium thermocellum* and *Caldicellulosiruptor* provide integrated platforms for enzyme expression, substrate degradation, and product synthesis at elevated temperatures [21,97]. The significance of these chassis is not simply to replace in vitro enzyme systems, but to place thermostable CAZymes within temperature-compatible cellular environments, allowing enzyme activity, substrate utilization, and metabolic conversion to be coordinated within the same system.

Overall, the core significance of synthetic biology-enabled thermophilic engineering is to establish an engineering interface between molecular-level thermostability improvement and high-temperature biomanufacturing. Rational design provides structural hypotheses, directed evolution enables experimental exploration, ancestral reconstruction offers stable scaffolds, and AI-assisted design provides predictive and generative capabilities. Synthetic biology organizes these strategies into workflows that can be built, tested, learned from, and deployed [96,98,99]. From this perspective, thermostable CAZymes are not merely improved enzymes, but programmable catalytic modules that can be combined, regulated, and embedded into biomanufacturing systems, as illustrated in Figure 3E [21,94,95]. No single engineering strategy is universally optimal. Structure-guided rational design is efficient when reliable structural and mechanistic information is available, but it may overlook long-range or epistatic effects. Directed evolution can identify nonintuitive solutions without complete mechanistic knowledge, although its effectiveness depends strongly on library quality and screening throughput. Consensus design and ancestral sequence reconstruction can provide stable starting scaffolds, but require sufficiently diverse homologous sequences and subsequent experimental validation. AI-assisted approaches can reduce the experimental search space, yet their performance depends on training-data quality and process-relevant validation, whereas synthetic biology is most useful when thermostability must be coordinated with expression, modular organization, host physiology, and process conditions. Because reported success rates depend on library size, assay sensitivity, and the definition of success, they are not directly comparable across studies. The experimental requirements, strengths, trade-offs, and best-use scenarios of these strategies are summarized in Table 1.

## 4. Thermophilic Amylases: A Successful Paradigm of High-Temperature Bioprocessing

The preceding sections discussed the design logic of thermostable CAZymes from the perspectives of molecular stability and engineering strategies. However, for industrial biocatalysis, enzyme thermostability is not an isolated molecular parameter but must be evaluated within specific substrates, processing windows, and application contexts. Different carbohydrate feedstocks possess distinct structural features and processing requirements, thereby imposing different demands on CAZyme stability, catalytic persistence, and process compatibility.

Starch processing represents one of the most mature industrial scenarios for high-temperature CAZyme applications. As an important storage polysaccharide and renewable carbon resource, starch is not only a major dietary carbohydrate, but also a key feedstock for producing glucose, maltose syrup, high-fructose syrup, fermentable sugars, and diverse food and bio-based products [11,109,110]. Compared with lignocellulose, which has a more complex architecture and requires highly coordinated multienzyme systems for deconstruction, starch has a relatively well-defined composition, processing workflow, and set of dominant catalytic functions. It therefore provides a mature model for understanding process-driven thermostable enzyme engineering. This section uses starch processing as a representative case to discuss the structural and catalytic basis of thermostable amylases, process-oriented engineering strategies, and their implications for high-temperature CAZyme design.

### 4.1. Process-Defined Thermal Demands in Starch Processing

Native starch mainly exists as granules composed of amylose and amylopectin. Amylose consists primarily of glucose units linked by α-1,4 glycosidic bonds, whereas amylopectin contains short α-1,4-linked chains with α-1,6 branch points, forming ordered cluster structures within starch granules [11,110]. In this granular architecture, some amylopectin chains pack into double helices and form crystalline regions, while branch points, more loosely arranged glucan chains, and part of the amylose are mainly distributed in amorphous regions. The alternating arrangement of crystalline and amorphous regions gives native starch poor solubility in cold water and limits the direct access of starch-converting enzymes to internal glycosidic bonds. Therefore, the thermal requirement in starch processing first arises from the physicochemical transformation of the substrate itself. During hydrothermal gelatinization, starch granules absorb water and swell, the ordered amylopectin structures are disrupted, and part of the amylose leaches out. As a result, the substrate changes from a compact granular state into a hydrated, swollen, highly viscous, and more enzyme-accessible form [11,109].

This gelatinized substrate state determines that subsequent starch conversion usually proceeds through a staged enzymatic process rather than a single hydrolytic step. In the liquefaction stage, α-amylase acts on high-temperature, swollen, and dynamically dispersed starch substrates, rapidly cleaving internal α-1,4 glycosidic bonds to reduce viscosity and generate dextrins [11,111]. In the saccharification stage, glucoamylases, debranching enzymes, and other starch-converting enzymes further hydrolyze dextrins and branched oligosaccharides, directing the products toward glucose, maltose syrup, or other target sugars [109]. In some product routes, subsequent isomerization further changes the sugar composition. Thus, thermostability in starch processing should be understood as stage-specific functional retention throughout the liquefaction–saccharification process. α-Amylases need to maintain an ordered catalytic groove, metal-ion-stabilized conformation, and rapid endo-cleavage activity under high-temperature, high-viscosity conditions with continuously changing substrate states [11,111]. Downstream saccharifying and debranching enzymes, in contrast, need to maintain catalytic adaptability under different pH values, temperatures, and product requirements [109,110].

The thermal demand in starch processing is therefore jointly defined by substrate state and process stage. For α-amylase, the most critical pressure arises from the liquefaction stage, where the enzyme must rapidly reduce viscosity and sustain continuous cleavage of internal α-1,4 glycosidic bonds in a hot, viscous, and dynamically dispersed substrate environment after gelatinization [109]. For saccharifying and debranching enzymes, thermostability is more reflected in their ability to maintain saccharification efficiency and product-profile control under downstream changes in temperature, pH, and product targets [11].

### 4.2. Structural and Catalytic Features of Thermophilic Amylases

In starch-converting enzyme systems, α-amylase occupies a special position because it is directly embedded in the liquefaction stage, where thermal demand is the strongest. After gelatinization, starch remains in a high-temperature, highly viscous, and dynamically dispersed state. α-Amylase must rapidly cleave internal α-1,4 glycosidic bonds under these conditions to reduce viscosity and generate dextrins. According to the classification of starch-converting enzymes by van der Maarel et al., α-amylase is a typical endoamylase responsible for initial starch depolymerization and viscosity reduction. In contrast, downstream enzymes such as glucoamylases, debranching enzymes, and transferases mainly act on liquefied dextrins or soluble oligosaccharides to promote saccharification, debranching, or the formation of specific products. Therefore, α-amylase provides a representative model for understanding structural stability, substrate recognition, and catalytic persistence during high-temperature starch liquefaction [112,113].

Typical α-amylases have a multidomain architecture composed of A, B, and C domains. Based on structural studies of bacterial and mammalian α-amylases, Nielsen and Borchert pointed out that the A domain forms a conserved (β/α)8-barrel and contains the key catalytic residues. The B domain protrudes from the A domain and contributes to the formation of the substrate-binding groove, while also being closely associated with metal-ion binding, substrate recognition, and stability [114]. The C domain is located at the C-terminus and mainly supports overall folding stability. This division of structural roles allows α-amylase to combine a conserved catalytic framework with a tunable substrate-binding region: the A domain maintains the catalytic core, whereas the B domain, loop regions, and A/B interface regulate substrate binding, product profile, metal dependence, and thermostability [112,114].

This structural organization is first reflected in substrate recognition. The acarbose-bound structure of *Bacillus subtilis* α-amylase resolved by Kagawa et al. showed that substrate-like oligosaccharides can occupy multiple sugar-binding sites along the active-site groove. This indicates that the active center of α-amylase is an open substrate-binding groove extending along the glucan chain. MacGregor et al. further proposed that the active sites of α-amylase family enzymes consist of multiple subsites, each interacting with one glucose residue, with glycosidic bond cleavage usually occurring between the −1 and +1 subsites. This multisite recognition mode enables α-amylase to bind internal segments of starch chains and perform endo-hydrolysis, thereby meeting the requirement for rapid viscosity reduction and initial starch depolymerization during liquefaction [115].

At the catalytic level, α-amylase relies on a highly conserved Asp/Glu/Asp catalytic triad to perform retaining glycosidic bond hydrolysis. Through mutational, kinetic, and structural analyses of human pancreatic α-amylase, Rydberg et al. clarified the functional roles of D197, E233, and D300: D197 acts as the catalytic nucleophile, E233 functions as the general acid/base catalyst, and D300 participates in organizing the catalytic conformation and positioning the water molecule. This reaction is generally described as a retaining double-displacement mechanism. The nucleophilic Asp first attacks the anomeric carbon to form a glycosyl–enzyme intermediate, after which Glu activates a water molecule to complete deglycosylation and product release. Thus, high-temperature catalysis by α-amylase requires not only stable global folding, but also the preservation of catalytic-residue geometry and substrate positioning under elevated temperatures [19,116].

Metal ions further connect thermostability with functional conformation. Machius et al. resolved the metal-binding structure of *Bacillus licheniformis* α-amylase and compared it with the metal-depleted apo form [114]. They found that the conserved Ca^2+^ site, together with an additional Ca^2+^ and a central Na^+^, forms a Ca–Na–Ca metal triad. Metal binding induces a neighboring 21-residue region to change from a disordered to an ordered state and contributes to one side of the extended substrate-binding site. Another Ca^2+^ is located at the A/C domain interface and may further reinforce overall structural stability [114]. These findings indicate that Ca^2+^ and Na^+^ in α-amylase do not merely increase protein rigidity; they also couple structural stability, substrate recognition, and catalytic competence by stabilizing the A/B interface and the substrate-binding groove [113,114].

**Figure 4:**
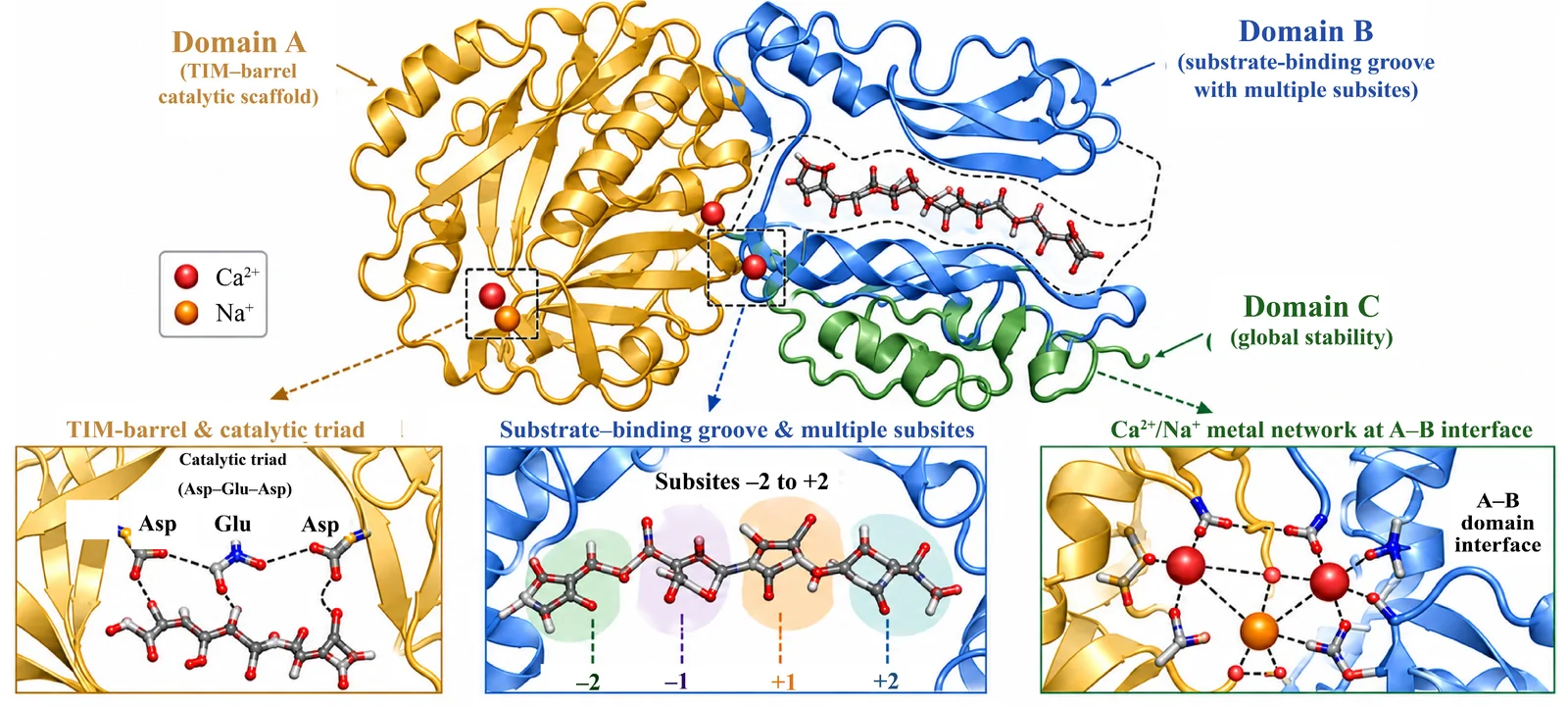
Representative structural features underlying α-amylase function and thermostability. The three-domain architecture of a typical α-amylase consists of a TIM-barrel catalytic domain A, an insertion domain B that shapes an open substrate-binding groove with multiple subsites, and a C-terminal domain C that contributes to global folding stability. The conserved Asp–Glu–Asp catalytic triad is embedded in the TIM-barrel scaffold, while Ca^2+^/Na^+^ metal-binding sites located at the A–B (and/or A–C) interface stabilise the extended binding groove and couple structural integrity with catalytically competent conformations at high temperature. This figure is a conceptual schematic summarizing representative structural and functional features of α-amylases. It is not derived from a specific PDB structure and does not depict precise atomic coordinates or spatial geometry.

Taken together, the function of α-amylase during high-temperature liquefaction depends on the coordinated maintenance of multiple structural features. The TIM-barrel provides a stable catalytic framework, the open substrate-binding groove supports internal recognition and endo-hydrolysis of starch chains, the Asp/Glu/Asp triad executes retaining glycosidic bond cleavage, and the metal-ion network preserves functional conformation at elevated temperatures [27,29]. Figure 4 schematically summarizes representative structural features associated with α-amylase catalysis and thermostability, rather than depicting a specific experimentally determined structure. This structure–catalysis basis also provides direct guidance for the subsequent engineering of thermophilic amylases [19,113].

### 4.3. Engineering Thermally Robust Amylases for Process Compatibility

Stabilization of flexible regions is one of the most direct strategies in structure-guided α-amylase engineering. Zhu et al. used α-amylase from *Geobacillus stearothermophilus* as a model and applied molecular dynamics simulations to identify flexible regions with increased fluctuations at elevated temperatures. Disulfide bonds were then rationally introduced into these local structural weak points to restrict loop conformational changes and improve local stability. The resulting mutant AmyS1 exhibited enhanced thermostability and showed improved substrate conversion during corn starch liquefaction at 100 °C. This case demonstrates that local flexible regions can serve as engineering entry points linking molecular thermostability to liquefaction performance. Other cases involving loop grafting or flexible-region engineering summarized in Table 2 further support this view [117].

Multipoint mutation reflects the cooperative stabilization of multiple structural nodes. Yuan et al. engineered α-amylase from *Bacillus amyloliquefaciens* by combining sequence alignment, homology modeling, and molecular docking to redesign several nonconserved regions. This work simultaneously increased the rigidity of loops in the B domain, strengthened interactions near Asp187 associated with the Ca–Na–Ca metal network, and improved local stability within the A domain, leading to the mutant MuBAA with enhanced thermostability and catalytic performance. This case indicates that high-temperature function in α-amylase is not determined by a single stabilizing element, but depends on the coordinated maintenance of the substrate-binding region, metal-ion coordination network, and TIM-barrel catalytic framework. The rational design cases of *Bacillus licheniformis* α-amylase listed in Table 2 reflect a similar principle: targeted site engineering can, to some extent, improve thermostability and catalytic activity simultaneously, rather than forcing a simple trade-off between the two [118].

**Table 2:** Representative engineering strategies for improving α-amylase thermostability.

Target	Engineering Strategy	Main Results	Reference
*Bacillus licheniformis* α-amylase	High-temperature MD simulation and FoldX-assisted rational design	E271L/N278K improved catalytic efficiency, whereas E336S exhibited a 1.43-fold longer inactivation half-life at 95 °C than WT.	[119]
*Geobacillus stearothermophilus* α-amylase	Rational disulfide-bond design in flexible regions	The mutant AmyS1 exhibited an approximately 40-fold longer half-life at 90 °C	[117]
*Bacillus amyloliquefaciens* α-amylase	Multipoint mutation targeting domain B loop and Ca^2+^-associated regions	The mutant MuBAA retained higher residual activity than WT at 50 and 60 °C, with a specific activity of 206.61 U/mg, 99.1% higher than that of the wild type.	[118]
*Bacillus licheniformis* α-amylase	Structure-guided rational design and site-directed mutagenesis	Q360C showed nearly threefold higher activity and retained 75% residual activity after pre-incubation at 70 °C for 30 min compared with 59% for WT.	[120]
*Geobacillus stearothermophilus* α-amylase	Computational loop grafting	Mutant had an 8.0-fold longer half-life at 100 °C with improved catalysis.	[120]
*Bacillus cereus* GL96 α-amylase	Computer-assisted directed evolution and site-directed mutagenesis	D162K showed 5 °C higher than optimum temperature and retained more than 40% of its initial activity up to 80 °C where the native lost its function.	[121]

Computationally assisted design further improves the precision of mutation-site selection. Fan et al. used *Bacillus licheniformis* α-amylase as a model and combined high-temperature molecular dynamics simulations with FoldX-based free-energy calculations to identify candidate sites located in flexible regions, distant from the catalytic triad, and favorable for reducing folding free energy. A small mutant library was then constructed for experimental validation. Compared with large-scale random screening, this strategy is more suitable for the fine optimization of mature industrial enzymes because it allows limited and precise modification of thermally sensitive regions while preserving the catalytic center and substrate-binding architecture. Other cases involving computationally assisted directed evolution and site-directed mutagenesis in Table 2 further indicate that α-amylase engineering is moving from empirical screening toward a more efficient route of computational prediction, small-library construction, and experimental validation [119].

Overall, the cases discussed in the text and the additional examples summarized in Table 2 show that thermostable α-amylase engineering has developed along several relatively clear directions. These include restricting local conformational fluctuations through disulfide bonds, loop grafting, or flexible-region engineering; cooperatively stabilizing the substrate-binding region, metal-ion network, and catalytic framework through multipoint mutation or interface reinforcement; and improving the efficiency of engineering-site selection through MD simulations, FoldX calculations, directed evolution, and small mutant libraries. These strategies share a common goal: to maintain substrate binding, catalytic geometry, and functional conformation of α-amylase under high-temperature, high-viscosity, and dynamically changing substrate conditions. Thus, α-amylase is not only a mature industrial enzyme for starch liquefaction, but also a clear example of how structural information can be translated into process-relevant CAZyme engineering.

## 5. Thermophilic Cellulases: Toward Programmable Lignocellulose Deconstruction

### 5.1. Thermal Challenges in Lignocellulose Conversion

Although both cellulose and starch conversion rely on multistep enzymatic processes, the origins of their thermostability requirements are fundamentally different. In starch processing, hydrothermal gelatinization can substantially disrupt native granular structures, converting the substrate into a more dispersed and enzyme-accessible state and thereby enabling subsequent liquefaction and saccharification. In contrast, cellulose remains largely insoluble, highly crystalline, and structurally heterogeneous even after pretreatment [122,123]. Therefore, cellulases must act for extended periods at the solid–liquid interface, where they carry out adsorption, chain disruption, glycosidic bond cleavage, product release, and multienzyme cooperation on substrate surfaces with limited accessibility [123].

The structural challenge of cellulose first arises from its highly ordered hierarchical organization. Native cellulose is composed of linear glucose chains linked by β-1,4 glycosidic bonds, which further assemble into elementary fibrils, microfibrils, and higher-order fibrous structures [122]. Intrachain and interchain hydrogen-bond networks, together with van der Waals interactions between adjacent sheets, generate tightly packed crystalline regions that strongly restrict enzyme access to the interior of microfibrils [122,123]. At the same time, native cellulose is not a completely uniform crystalline material. It also contains amorphous regions, chain dislocations, microfibril twisting, pores, and local defects that provide limited accessible sites. The coexistence of high crystallinity and local heterogeneity means that cellulose hydrolysis mainly occurs on constrained solid surfaces rather than in a homogeneous soluble phase [123].

**Figure 5:**
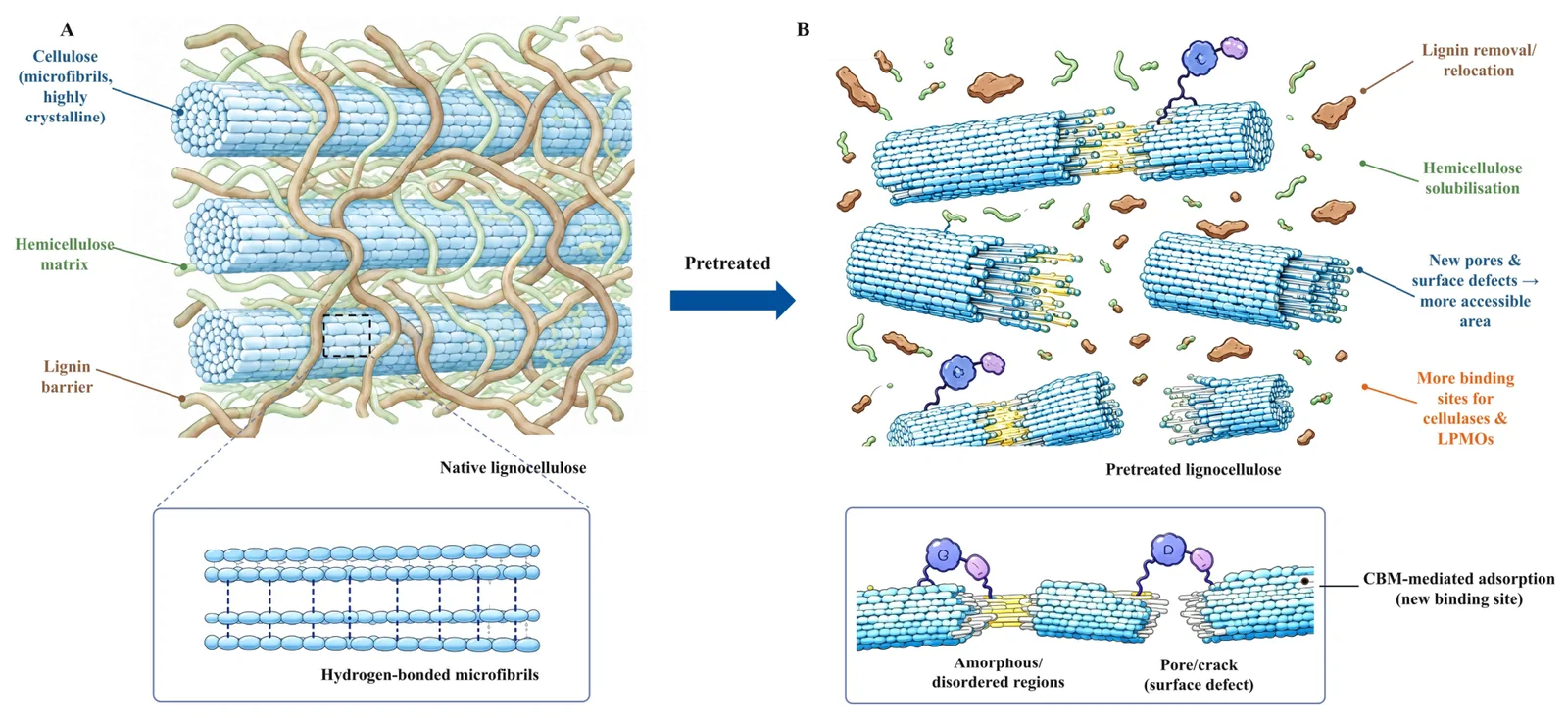
Substrate complexity and accessibility in cellulose deconstruction. (**A**) Native lignocellulose shows tightly packed cellulose microfibrils embedded in a hemicellulose–lignin matrix. Extensive intra- and interchain hydrogen bonding and limited porosity restrict enzyme access to only a small fraction of cellulose surfaces. (**B**) Pretreatment (e.g., steam explosion, dilute-acid) partially removes or redistributes lignin, solubilises hemicellulose, and generates amorphous regions, cracks, and pores, thereby increasing the enzyme-accessible surface area of cellulose. Despite these changes, cellulose remains largely insoluble and heterogeneous, so thermostable cellulases must sustain adsorption, chain capture, and processive hydrolysis at solid–liquid interfaces under high-temperature, high-solids conditions.

Thermostability in cellulose hydrolysis should therefore not be understood only as the ability of enzymes to resist thermal unfolding. For cellulases, high-temperature performance depends more directly on whether the enzyme can continuously maintain substrate adsorption, chain capture, processive cleavage, and multienzyme synergy at restricted solid interfaces [122,123]. Compared with starch liquefaction, the key thermal challenge in cellulose deconstruction is not only to preserve catalytic activity, but also to maintain effective enzyme–substrate interactions on insoluble, crystalline, and structurally heterogeneous substrate surfaces. The contrasting structural organization and enzyme accessibility of native and pretreated lignocellulose are illustrated in Figure 5A,B, respectively.

### 5.2. Modular Architecture and Cooperative Catalysis of Thermostable Cellulases

The low accessibility of cellulose makes cellulase catalysis highly dependent on modular architecture [41,124]. Many efficient cellulases contain not only a catalytic domain, but also a carbohydrate-binding module (CBM) and a linker [23,37,125]. The CBM enriches and positions enzyme molecules on the cellulose surface, while the linker provides spatial flexibility between the binding module and the catalytic domain, allowing the catalytic domain to access locally exposed cellulose chains or to move processively along the chain during hydrolysis [23,37]. Unlike α-amylase, which mainly recognizes relatively dispersed starch substrates through an open active-site groove, cellulases rely more heavily on surface targeting, modular cooperation, and sustained catalysis at solid interfaces [41,124].

**Figure 6:**
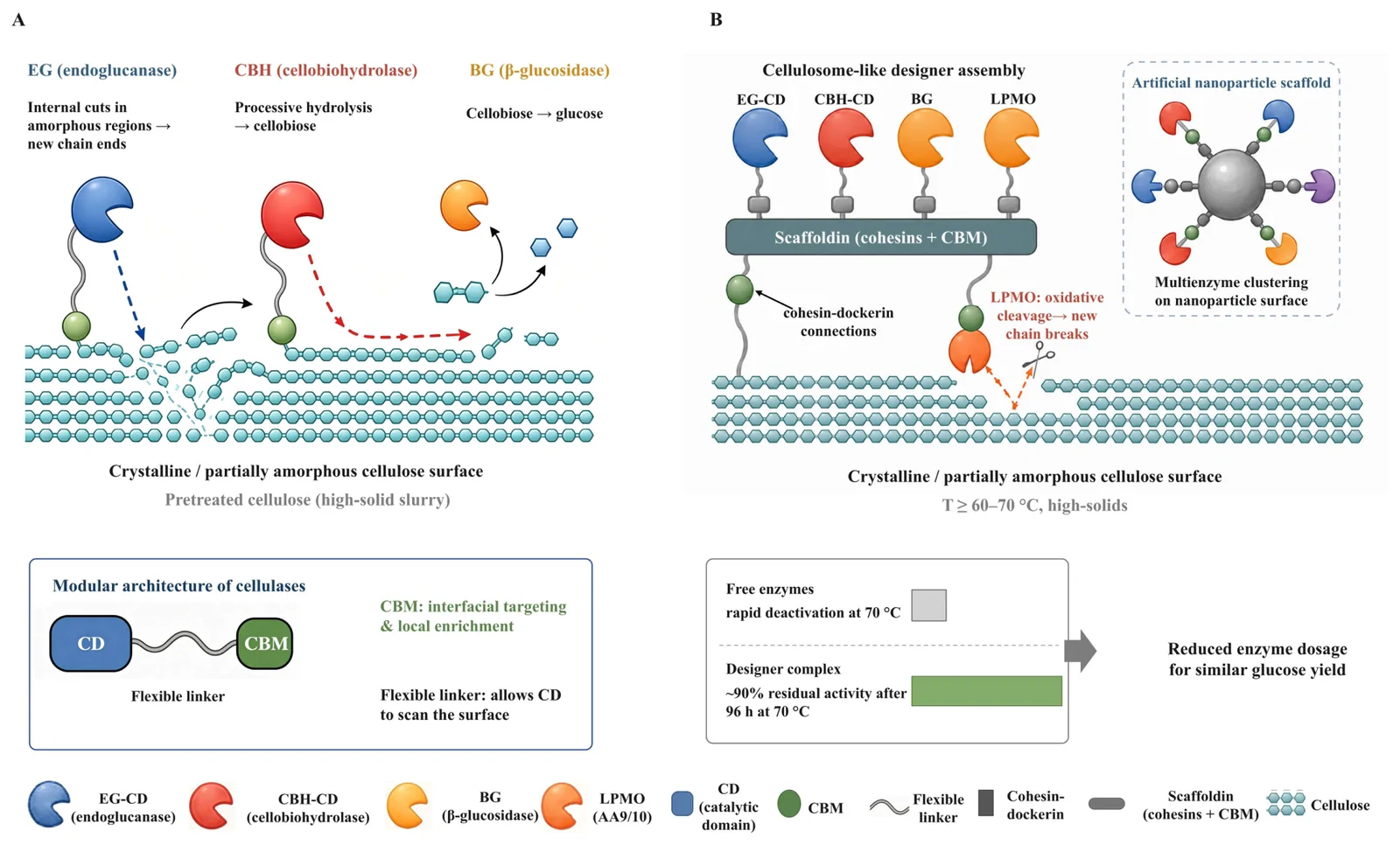
Modular and cooperative mechanisms of thermophilic cellulase systems. (**A**) In free-enzyme systems, individual thermophilic endoglucanases (EGs), cellobiohydrolases (CBHs) and β-glucosidases (BGs) act at the solid–liquid interface. CBMs mediate adsorption and local enrichment on heterogeneous cellulose surfaces, EGs introduce internal cuts in amorphous regions to create new chain ends, CBHs processively release cellobiose from exposed chain ends, and BGs convert cellobiose to glucose, relieving product inhibition. (**B**) In cellulosome-like complexes and artificial multienzyme assemblies, scaffoldins, cohesin–dockerin interactions, or nanoparticle scaffolds cluster multiple catalytic domains and CBMs in close proximity on the cellulose surface. Such spatial organisation enhances interfacial targeting, processive hydrolysis, and high-temperature stability, allowing reduced enzyme loading and improved sugar yields compared with free enzyme mixtures. In the schematic representation, the central gray sphere denotes the scaffold or core, the connecting elements represent linkers, and the differently colored peripheral symbols represent distinct functional modules assembled on the scaffold.

Cellulose hydrolysis also requires functional division among different enzyme components [126,127]. Endoglucanases usually act preferentially on amorphous regions or locally accessible chain segments, introducing internal cuts into cellulose chains and generating new chain ends. Cellobiohydrolases then start from these chain ends and processively release cellobiose along the cellulose chain. β-Glucosidases further hydrolyze cellobiose and short cello-oligosaccharides into glucose, thereby relieving product inhibition of upstream hydrolytic enzymes by cellobiose. Thus, cellulose hydrolysis is composed of chain opening, processive chain-end depolymerization, and product removal (Figure 6A). Under high-temperature conditions, what must be maintained is the coordination among these consecutive steps, rather than the transient activity of a single enzyme [124].

Accessory enzymes further expand the cooperative mechanisms of cellulose deconstruction [41,126]. Lytic polysaccharide monooxygenases (LPMOs) can act oxidatively on exposed cellulose surfaces, introducing chain breaks and local defects into crystalline surface layers and thereby improving the efficiency of subsequent endoglucanase and cellobiohydrolase action. In this context, the significance of LPMOs is not to replace classical hydrolytic enzymes, but to show that efficient cellulose deconstruction often requires hydrolytic and oxidative mechanisms to jointly improve substrate accessibility [127].

At the system level, microbial cellulose degradation is mainly organized through two strategies: free-enzyme systems and cellulosome systems [30,128]. In free-enzyme systems, different cellulases independently diffuse to the cellulose surface and achieve dynamic cooperation through their own catalytic domains, CBMs, and linkers. In cellulosome systems, scaffoldins, cohesin–dockerin interactions, and CBMs organize multiple catalytic subunits into spatially concentrated multienzyme complexes (Figure 6B) [30,129,130,131]. The former emphasizes dynamic cooperation of enzyme components on the substrate surface, whereas the latter emphasizes spatial proximity and substrate targeting of multiple enzyme components. Therefore, the performance of thermophilic cellulases depends not only on whether the catalytic domain remains folded at high temperature, but also on whether CBM-mediated adsorption, linker-supported spatial mobility, processive hydrolysis, and multienzyme spatial organization can be continuously maintained at high-temperature solid interfaces [41,124,125,128].

### 5.3. Engineering Thermostable Cellulases for High-Temperature Cellulose Deconstruction

Because cellulose hydrolysis has a strong interfacial-catalysis character, the engineering of thermophilic cellulases cannot focus only on a single catalytic domain. Unlike α-amylase engineering in starch liquefaction, which mainly emphasizes catalytic-groove stability, metal-ion-dependent conformation, and high-temperature endo-cleavage activity, cellulase engineering must also consider processive catalytic domains, CBM-mediated substrate binding, linker flexibility, auxiliary oxidative activity, and multienzyme spatial organization. The representative cases below illustrate three engineering levels: mining native thermophilic templates, engineering interfacial binding, and constructing artificial spatial organization.

Mining natural thermostable templates from thermophilic environments is an important route to obtain cellulases suitable for high-temperature operation. Takeda et al. identified a GH6 family cellobiohydrolase, HmCel6A, from a metagenomic library derived from Japanese hot spring sediments and further characterized the natural variant HmCel6A-3SNP. This enzyme showed an optimal reaction temperature of 95 °C and a T_m_ of 96 °C in the presence of Ca^2+^, indicating strong potential for high-temperature cellulose saccharification. Structural analysis showed that HmCel6A-3SNP retains the typical architecture of GH6 cellobiohydrolases, including a catalytic barrel, conserved catalytic residues, and an active-site tunnel. It also contains hydrophobic clusters, charge interactions, Ca^2+^-binding sites, disulfide bonds, and shortened substrate entrance and exit loops. This case suggests that the high-temperature performance of processive cellulases depends not only on global folding stability, but also on the coordinated maintenance of the active-site tunnel, loop dynamics, and chain-threading capability.

Interfacial binding engineering represents another important direction. DeChellis et al. used the GH5 endoglucanase Cel5A from *Thermobifida fusca* and its native family 2a CBM as a model to examine how module surface charge affects cellulose binding, thermostability, and biomass hydrolysis performance. Instead of directly modifying the conventional catalytic center, this study used Rosetta to design supercharged variants with different net-charge combinations, thereby tuning the surface charge distribution of the CBM and catalytic domain. Some variants carrying CBM-only modifications showed markedly improved hydrolytic activity on pretreated corn stover, crystalline cellulose, and amorphous cellulose. The best-performing CBM variants exhibited approximately 2–5-fold higher activity than the wild type, and some variants showed a 5–10 °C increase in optimal hydrolysis temperature. These results indicate that CBMs are not merely auxiliary binding appendages. Their surface properties can independently regulate adsorption strength, desorption behavior, and catalytic turnover on cellulose surfaces, making them effective engineering targets for improving the conversion of complex substrates.

A further engineering level is the regulation of cellulase-module spatial organization. Nakazawa et al. constructed hybrid cellulase complexes by artificially assembling catalytic domains and cellulose-binding domains. In this study, 49 biotinylated catalytic domains and 30 biotinylated cellulose-binding domains were selected from databases, and different catalytic domain–cellulose-binding domain (CD–CBD) modules were clustered onto streptavidin-modified nanoparticles to mimic substrate targeting, enzyme proximity, and synergistic catalysis in natural cellulosomes. Screening showed that the best CD–CBD combinations did not necessarily correspond to natural pairings; some cross-origin combinations instead exhibited higher hydrolytic performance. Further combinatorial optimization also increased the production of saccharification products. More importantly, the artificial complex showed greatly enhanced thermostability. Free enzymes lost most of their activity after incubation at 70 °C for 6 h, whereas the optimized hybrid complex retained approximately 90% of its initial activity after incubation at 70 °C for 96 h. This demonstrates that cellulase thermostability can be improved not only by stabilizing individual domains, but also by nanoparticle-mediated module clustering and cellulosome-like spatial organization.

In addition to these representative cases, other studies summarized in Table 3 further expand the engineering levels of thermophilic cellulases, including catalytic-domain stabilization, β-glucosidase engineering, LPMO stabilization, module/domain recombination, and designer cellulosome construction. Together, these cases show that cellulase thermostability engineering is moving beyond the stabilization of single-enzyme folding toward the integrated regulation of substrate binding, auxiliary oxidation, relief of product inhibition, and multienzyme spatial organization.

## 6. Conclusions and Outlook

**Figure 7:**
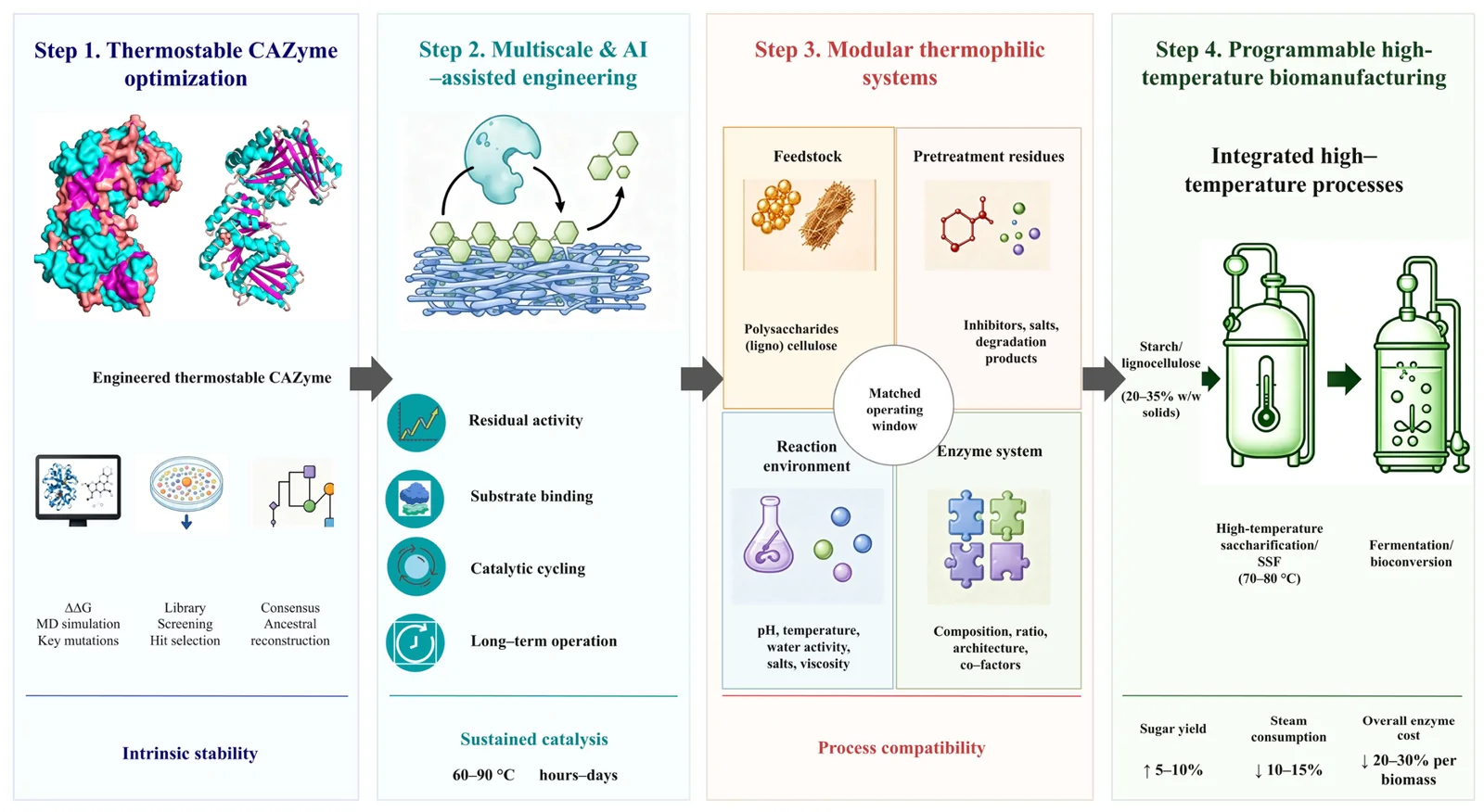
From thermostable CAZyme optimization to programmable biomanufacturing systems.

Thermostable CAZymes are key catalytic modules that connect complex carbohydrate substrates with accessible sugar streams, platform intermediates, and cell factories in high-temperature biomanufacturing. This review shows that CAZyme thermostability should be understood as the integrated ability of enzymes to maintain functional conformation and catalytic capacity under target temperatures, real substrates, inhibitors, multienzyme systems, and process windows. Molecular stabilizing elements, conformational dynamics, modular architecture, and system compatibility jointly determine whether a thermostable enzyme can be translated into practical process performance. In recent years, rational design, directed evolution, ancestral sequence reconstruction, AI-assisted modeling, and synthetic biology have been driving thermostable CAZyme engineering from empirical modification toward more predictable, iterative, and process-aware design [25,54,78,106]. Future efforts should further integrate sequence–structure prediction with real high-temperature phenotypic screening, develop standardized thermophilic chassis and DBTL platforms, and directly evaluate the long-term function of engineered enzymes in starch conversion, lignocellulose deconstruction, and other carbohydrate-based biomanufacturing processes. For high-temperature industrial environments, the goal of thermostable CAZyme engineering is no longer simply to obtain a “heat-resistant” single enzyme, but to construct robust enzyme systems capable of stably driving efficient renewable-carbon conversion [107,132]. This progression from molecular-level stabilization to system-level deployment is summarized in Figure 7.

## Figures and Tables

**Table 1:** Engineering strategies of thermostable enzymes.

Strategy	Requirements	Strengths	Trade-Offs	Best Suited for	Representative Studies
Rational design	Structure/model; focused validation	Small libraries; interpretable	Prediction errors; local bias	Defined instability sites	[33,56,100,101]
Directed/semi-rational evolution	Variant libraries; high-throughput screening	Mechanism-independent; captures epistasis	Screening-intensive; low hit rate	Unclear mechanisms; complex traits	[102,103,104,105]
Consensus design/ASR	Homologous sequences; alignment/phylogeny	Stable scaffolds; structure optional	Sequence bias; possible function shifts	Homolog-rich families; unstable templates	[106]
AI/ML-assisted design	Training data/models; iterative validation	Rapid prioritization; reduced screening	Data-dependent; limited extrapolation	Large sequence spaces; DBTL cycles	[78]
Synthetic biology/system engineering	Modular constructs; host/process testing	System-level optimization; scalable iteration	Platform-intensive; host-dependent	Multienzyme and thermophilic systems	[37,107,108]

**Table 3:** Representative cases of thermostable cellulases and cellulase systems for high-temperature cellulose deconstruction.

Case	Strategy	Performance Metric	Implication	Reference
Glycosylated hyperthermostable designer cellulosome	Glycosylation and *C. bescii* as expression host	Glycosylated scaffoldin and enzyme components improved long-term stability at 75 °C and enhanced cellulose hydrolysis	Post-translational modification serves as a system-level strategy for stabilizing multienzyme cellulase complexes	[132]
TtCel7A native thermophilic enzyme	Discovery and characterization of native thermophilic multifunctional enzyme	Cellulase and xylanase activity half-lives decreased from 140 to 41 min and from 24 to 1.4 h, respectively, as the temperature increased from 50 to 70 °C	Natural thermophilic enzymes remain important sources for high-temperature biomass conversion	[133]
*Thermobifida fusca* Cel6A variants	Domain engineering and MD-assisted analysis	The cel6A.CBC variant showed enhanced activity on filter paper, alkali-treated bagasse and retained 62% activity after 60 min at 70 °C	Domain engineering can improve cellulase activity and thermostability on real or semi-real cellulose substrates	
Supercharged *Thermobifida fusca* Cel5A–CBM2a variants	Surface charge engineering of CBM–cellulase constructs	Negatively supercharged variants showed 3–4-fold higher activity, whereas some positively charged variants exhibited reduced binding or stability	Charge engineering needs to balance cellulose binding, catalytic activity and thermal stability	[134]
GH5 endoglucanase chimeras from mesophilic SoCel5 and thermophilic TeEgl5A	Structure and MD-guided chimera design	H8 and H9 increased T_50_ by 15–19 °C, T_m_ by 16.5–22.9 °C, and half-life at 55 °C by about 240- to 650-fold	Chimera engineering can transfer thermostability from thermophilic enzymes to mesophilic scaffolds.	[135]
Hyperthermophilic Endoglucanase Cel12B from Thermotoga maritima	Rational site-directed mutagenesis	Two recombinant enzymes showed improvement in activity by 77% and 87%, and retained 80% and 90.5% of initial activity after incubation at 80 °C for 8 h	Rational mutation can improve activity while preserving hyperthermostability	[136]

## Data Availability

No new data were created or analyzed in this study. Data sharing is not applicable to this article.

## Abbreviations

The following abbreviations are used in this manuscript:

DBTL	Design–build–test–learn
AI	Artificial intelligence
ASR	Ancestral sequence reconstruction
CAGR	Compound annual growth rate
CAZymes	Carbohydrate-active enzymes
CBD	Cellulose-binding domain
CBM	Carbohydrate-binding module
CD	Catalytic domain
DNA	Deoxyribonucleic acid
FACS	Fluorescence-activated cell sorting
GH	Glycoside hydrolase
ISM	Iterative saturation mutagenesis
LPMO	Lytic polysaccharide monooxygenase
MD	Molecular dynamics
ML	Machine learning
NDK	Nucleoside diphosphate kinase
PROSS	Protein Repair One-Stop Shop
TIM	Triosephosphate isomerase
WT	Wild type
